# Intravascular heavy chain-modification of hyaluronan during endotoxic shock

**DOI:** 10.1016/j.bbrep.2018.12.007

**Published:** 2018-12-26

**Authors:** Kevin Ni, Amar Gill, Danting Cao, Kengo Koike, Kelly S. Schweitzer, Stavros Garantziotis, Irina Petrache

**Affiliations:** a Department of Medicine, National Jewish Health, 1400 Jackson Street, Denver, CO 80206, USA; b Department of Biochemistry and Molecular Biology, Indiana University School of Medicine, Indianapolis, IN 46202, USA; c National Institute of Environmental Health Services, Durham, NC 27709, USA; d Department of Medicine, University of Colorado School of Medicine, Aurora, CO 80045, USA

**Keywords:** ALI, acute lung injury, AM, alveolar macrophage, CXCL2, chemokine (C-X-C motif) ligand 2, ECM, Extracellular matrix, HA, hyaluronic acid (hyaluronan), hAM, human alveolar macrophages, HC, heavy chain (IαI), hTSG-6, human TSG-6, IαI, inter-alpha-inhibitor, kDa, kiloDalton, LPS, lipopolysaccharide, Mega-Da, megaDalton, PαI, Pre-α-inhibitor, TBW, total body weight, TNFα, tumor necrosis factor α;, TSG-6, TNFα-stimulated gene-6, Hyaluronic acid, Inter-alpha-inhibitor, Serum-derived hyaluronan-associated protein, TNFα stimulated gene 6, Endotoxic shock

## Abstract

During inflammation, the covalent linking of the ubiquitous extracellular polysaccharide hyaluronan (HA) with the heavy chains (HC) of the serum protein inter alpha inhibitor (IαI) is exclusively mediated by the enzyme tumor necrosis factor α (TNFα)-stimulated-gene-6 (TSG-6). While significant advances have been made regarding how HC-modified HA (HC-HA) is an important regulator of inflammation, it remains unclear why HC-HA plays a critical role in promoting survival in intraperitoneal lipopolysaccharide (LPS)-induced endotoxemia while exerting only a modest role in the outcomes following intratracheal exposure to LPS. To address this gap, the two models of intraperitoneal LPS-induced endotoxic shock and intratracheal LPS-induced acute lung injury were directly compared in *TSG-6* knockout mice and littermate controls. HC-HA formation, endogenous TSG-6 activity, and inflammatory markers were assessed in plasma and lung tissue. *TSG-6* knockout mice exhibited accelerated mortality during endotoxic shock. While both intraperitoneal and intratracheal LPS induced HC-HA formation in lung parenchyma, only systemically-induced endotoxemia increased plasma TSG-6 levels and intravascular HC-HA formation. Cultured human lung microvascular endothelial cells secreted TSG-6 in response to both TNFα and IL1β stimulation, indicating that, in addition to inflammatory cells, the endothelium may secrete TSG-6 into circulation during systemic inflammation. These data show for the first time that LPS-induced systemic inflammation is uniquely characterized by significant vascular induction of TSG-6 and HC-HA, which may contribute to improved outcomes of endotoxemia.

## Highlights

• HC-HA deficiency accelerated mortality after IP LPS, but only modestly affected IT LPS outcomes.
• Both intratracheal (IT) and intraperitoneal (IP) LPS triggered lung HC-HA formation.
• IP LPS, but not IT LPS instillation induced intravascular TSG-6 and HC-HA.
• Intravascular HC-HA formation may be protective against LPS-induced injury.

## 1 Introduction

Hyaluronan (HA) is a linear polysaccharide that at baseline lacks covalent modifications (sulfation and proteoglycan core protein) characteristic of other members of the extracellular glycosaminoglycan family. However, HA fragments can be covalently modified with the heavy-chains (HC) of the serum protein inter-alpha-inhibitor (IαI), which primarily occurs during inflammation and inflammation-like processes such as ovulation [1]. This only known covalent modification of HA, is exclusively mediated by the secreted protein tumor necrosis factor α (TNFα)-stimulated gene-6 (TSG-6) [1], [2], an enzyme that is evolutionarily conserved in all vertebrates [3], [4]. HC-modified HA (HC-HA) formation improves survival outcomes in endotoxemic sepsis [5], [6], associated with retention of neutrophils within liver sinusoids [7], [8], [9]. However, HC-HA exerted only modest effects against localized intratracheal (IT) endotoxic exposures that cause acute lung injury (ALI) [10], despite rapid HC-modification of HA that paralleled the kinetics of lung inflammation. It remains unclear why HC-HA has a distinct impact depending on the route of lipopolysaccharide (LPS) exposure. Using *TSG-6* knockout (KO) mice, we tested the hypothesis that the significant protective effect of TSG-6 during systemic exposure to LPS is associated with more robust formation of intravascular HC-modified HA, which may be required to control outcomes of inflammation.

## 2 Materials and methods

### 2.1 Reagents

All materials and reagents were obtained from ThermoFisher (Waltham, MA, USA), unless otherwise specified.

### 2.2 Animal experiments

All animal experiments were approved by Institutional Animal Care and Use Committee at National Jewish Health. *TSG-6*-KO mice (BALB/c background) were originally created by Dr. Katalin Mikecz [2]. *TSG-6*-KO was confirmed by genotyping and demonstration of inability to form HC-modified HA, using a method previously described [2], [10]. Studies were conducted using sex- (male and female) and age matched (8–12 weeks) *TSG-6*-KO mice and wild type (WT) and heterozygous (HT) littermate controls.

#### 2.2.1 Mouse endotoxic shock model

*E. coli* O111:B4 LPS (L2630, MilliporeSigma, Burlington, MA, USA) was administered at a dose of 20 mg/kg body weight as a solution of 1.7 mg/mL in phosphate buffered saline (PBS) [5] injected *via* intraperitoneal (IP) route in the right lower quadrant. Survival was assessed every 6 h. For lung tissue and plasma analysis, mice were harvested at either 8 h or 12 h post LPS administration, as specified.

#### 2.2.2 Mouse intratracheal LPS-induced ALI model

*E. coli* O55:B5 LPS (L2880, MilliporeSigma) was administered at a dose of 20 μg LPS in 50 μL phosphate buffered saline (PBS) that was intratracheally (IT) instilled as described before [10]. Mouse weight was assessed daily, for up to four days.

#### 2.2.3 Plasma and perfused lung collection

Mice were euthanized by isoflurane overdose and bilateral thoracotomy. Whole blood was collected *via* puncture of the right ventricle using 1 mL syringe and needle filled with 100 μL concentrated sodium citrate (S5770–50 mL, MilliporeSigma). Lungs were then perfused with 10 mL of blood bank saline and snap frozen in liquid nitrogen. Whole blood was centrifuged (2000*g*, 10 min, 12 °C) to obtain plasma supernatant.

### 2.3 Measurements of messenger RNA (mRNA)

Total ribonucleic acid (RNA) was extracted from whole lung as previously described [10]. 1000–2000 ng of extracted RNA was used for complementary DNA production (High-Capacity cDNA Reverse Transcription). Real-time quantitative polymerase chain reaction (qPCR) was run on StepOnePlus and prepared using Taqman Universal PCR Master Mix and the following Taqman probe: *msTNFα* (Mm00443258_m1). Relative mRNA expression was calculated using the double delta comparative (ΔΔCt) method and *18 s* RNA loading control (Taqman Hs99999901_s1).

### 2.4 Measurement of HC-HA levels

Lung tissue was homogenized and treated with 1 U of *Streptomyces hyaluronlyticus* hyaluronidase (389561, MilliporeSigma) or PBS control, as previously described [10]. SDS-PAGE and western blot was performed on the samples as previously described [10] using rabbit-anti-hIαI antibody (A0301, DAKO, Agilent, Santa Clara, CA, USA), which has been validated for detecting mouse IαI and HC-HA in various mouse tissues [2], [11]. 7.5% Criterion TGX Stain-Free gels (Biorad, Hercucles, CA, USA) were imaged for total protein using ChemiDoc MP (Biorad). Densitometry was calculated using Image Studio Lite (Licor, Lincoln, NE, USA).

Plasma (40 μL) was treated with 1 U of *Streptomyces hyaluronlyticus* hyaluronidase or PBS control for 2 h at 37 °C and then 2 h at room temperature with mechanical agitation. Following addition of Laemmli buffer, SDS-PAGE and western blot with IαI antibody were performed as previously described [10].

### 2.5 TSG-6 activity assay

Endogenous TSG-6 activity in plasma was measured as described [12] with minor modifications. Plasma (40 μL) samples were mixed with 3 μg of 10-oligosaccharide HA (HYA-OLIGO10EF-1, Hyalose AMSBIO, Cambridge, MA, USA) for 2 h at 37 °C and then 2 h at room temperature with mechanical agitation. For positive controls, 20 ng recombinant human TSG-6 (R&D Systems, Minneapolis, MN, USA) was added to plasma. To generate the negative controls, ethylenediaminetetraacetic acid (EDTA) was mixed with plasma at a final concentration of 0.1 M before adding 10-oligosaccharide HA, because TSG-6 activity depends on divalent metal cations Ca^2+^ and Mg^2+^ [13], [14], [15]. Following addition of Laemmli buffer, samples were subjected to SDS-PAGE and western blotting as previously described [10], using an anti-IαI antibody which can detect HC covalently linked to 10-oligosaccharide HA [12].

### 2.6 Flow cytometry analysis of plasma

Whole blood was collected similarly as described above, but instead of sodium citrate, 100 μL of 0.5 M EDTA was used as anticoagulant. Anticoagulated whole blood (100 μL) was mixed with 900 μL of flow wash buffer (PBS with 9% FBS and 0.5 mM EDTA) and centrifuged (450*g*, 4 °C) to remove the supernatant. Remaining packed cells were diluted with 1 mL red blood cell lysis buffer (Pharm Lyse, Becton Dickinson or BD Biosciences, Franklin Lakes, NJ, USA) and pipetted up and down to lyse red blood cells. 1 mL of flow wash buffer and 5 mL of PBS was added to dilute the lysed suspension and centrifuged. Supernatant was discarded and the cell pellet was blocked with CD16/CD32 (clone 93, eBioscience) and stained with TER-119 (Biolegend, San Diego, CA, USA), CD45 (30-F11, BD Biosciences), Ly6G (1A8, Biolegend), CD11b (M1/70, eBioscience), Siglec-F (E50–2440, BD Biosciences), CD115 (AFS98, eBioscience). Flow data, which included running a minimum of 20,000 CD45^+^ leukocyte events for each sample, were collected using LSR II cytometer (BD Biosciences) and analyzed using Flowjo software (Ashland, Oregon, USA).

### 2.7 TNFα ELISA

Mouse plasma TNFα levels were determined using mouse TNFα DuoSet ELISA (R&D Systems), following the manufacturer's protocol. Plasma collected from IP LPS mice was diluted 1:5 with reagent diluent (1% BSA in PBS). Capture antibody was coated on Nunc MaxiSorp 96-well plates using 0.2 M BupH sodium bicarbonate buffer (pH 9.4).

### 2.8 Cell culture

Primary human lung microvascular endothelial cells (HMVEC-L) were obtained from Lonza (Allendale, NJ, USA) and cultured in microvascular endothelial growth medium (EGM-2-MV) (Lonza) following manufacturer's instructions. Cells were used for experiments between passages 6–7. Cells were treated for 24 h in EGM-2-MV with vehicle (0.1% BSA in PBS), 20 ng/mL TNFα (R&D Systems), 20 ng/mL IL1β (R&D Systems), or 20 ng/mL ultrapure *E. coli* LPS (LPS-EK, InvivoGen, San Diego, CA, USA). After collecting conditioned media supernatant (1000 *g*, 10 min spin), cells were rinsed once with PBS and then lysed for RNA extraction.

Primary human alveolar macrophages (hAM) culture were obtained and cultured as described previously [10]. Cells were stimulated for 20 h with vehicle (0.1% bovine serum albumin-BSA in PBS), 20 ng/mL TNFα, or 50 ng/mL ultrapure *E. coli* LPS (LPS-EK).

### 2.9 hTSG-6 western blot and ELISA

Western blotting for hTSG-6 was described previously [10]. Briefly, goat-anti-human TSG-6 (AF2104, R&D Systems) was used to probe for hTSG-6 and hTSG-6-HC, which readily forms in the presence of fetal bovine serum (FBS) and is absent when FBS is omitted [10]. 4–20% gradient Criterion TGX Stain-Free gels were imaged for total protein using ChemiDoc MP. HMVEC-L conditioned media was concentrated four-fold using 10 kDa cutoff centrifugal filters (Microcon-10, MilliporeSigma).

hTSG-6 levels in conditioned media was measured by highly sensitive and siRNA validated sandwich ELISA that was described previously [10], [16]. hTSG-6 standard curve was prepared using FBS to match the FBS content in conditioned media, because the formation of TSG-6-HC covalent intermediate interferes with sandwich ELISA detection [10]. HMVEC-L conditioned media was concentrated eight-fold using 10 kDa cutoff centrifugal filters described above.

### 2.10 Statistical analyses

Data were analyzed using ANOVA with Tukey's multiple comparison testing using Graphpad Prism (Graphpad Software, La Jolla, CA, USA). Data points in graphs signify individual mice or independent experiments, unless otherwise specified. Results were considered significant at *P* < 0.05.

## 3 Results

### 3.1 Effect of TSG-6 on survival during severe endotoxemia

**Fig. 1:**
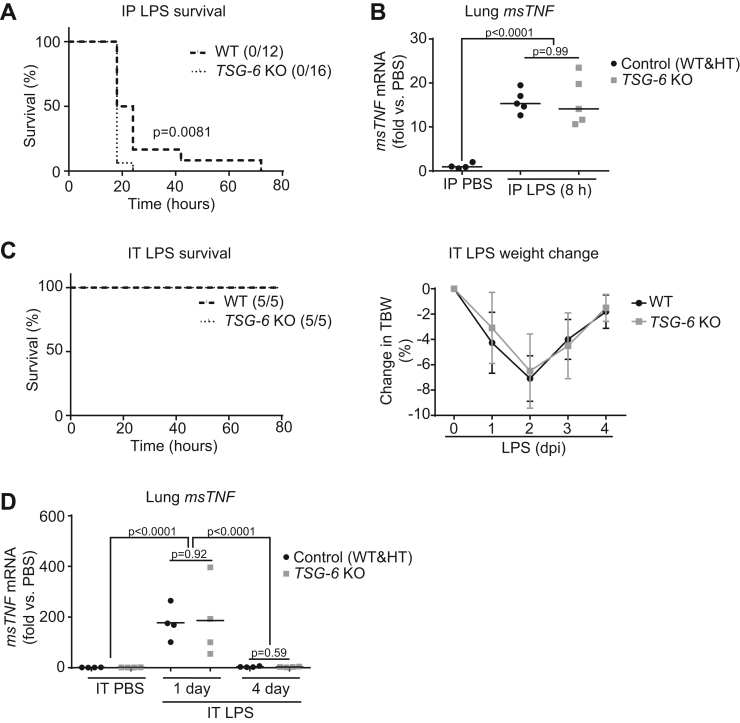
**Effect of TSG-6 on survival during endotoxic shock. A.** Survival was assessed over 3 days after intraperitoneal (IP) LPS (20 mg/kg total body weight or TBW) administration. *n* = 12–16 mice per treatment group. Analyzed with Log-rank (Mantel-Cox test). **B**. Expression of *msTNFα* was assessed by qPCR in whole lung from *TSG-6* KO and control (WT & HT) littermate lungs were assessed at 8 h following IP LPS injury. **C**. Survival and daily TBW change over four days (mean +/- SD, % change from baseline) were assessed following intratracheal (IT) LPS (20 μg/mouse) instillations. **D**. Expression of *msTNFα* in whole lung from *TSG-6* KO and control lungs at the indicated timepoints following IT LPS injury. *n* = 4–6 mice per treatment group. Data analyzed with ANOVA and Tukey's multiple comparisons. Wildtype (WT); Heterozygote (HT); Knockout (KO); days post injury (dpi).

Consistent with previous reports [5], [6], we observed that *TSG-6* knockout (KO) mice had a more rapid onset of mortality following systemic LPS administration (20 mg/kg weight; IP) with 18 h median survival, compared to wild type (WT) mice that had 21 h median survival (Fig. 1**A**). As previously reported [17], systemic endotoxemia was associated with increased *TNFα* expression in the lung tissue (Fig. 1**B**) consistent with lung injury, that preceded the onset of mortality and was similar in WT and KO mice. *TSG-6* KO mice that were exposed to LPS *via* intratracheal (IT) instillation exhibited similar survival outcomes and loss and recovery of total body weight as WT littermates (Fig. 1**C**). Following IT LPS instillation, but preceding the peak of weight loss, we noted marked *TNFα* induction which was also similar in WT and KO mice (Fig. 1**D**). These results suggest that the HC-HA effect on the outcomes of injury is dependent on the route of endotoxic exposure, which may be linked to a distinct localization of HC-HA formation.

### 3.2 Lung HC-HA during severe endotoxemia

**Fig. 2:**
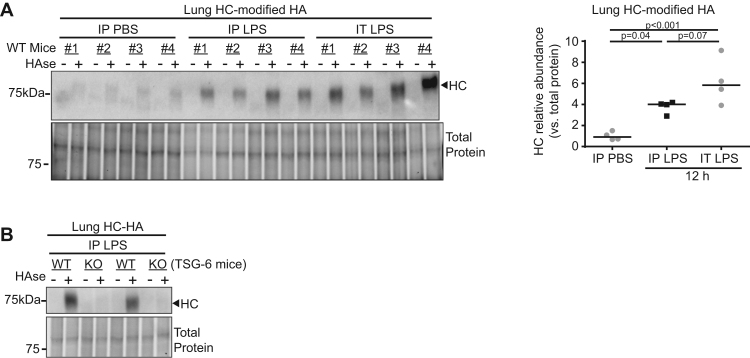
**Lung parenchymal HC-HA formation after IP or IT LPS injury**. Abundance of heavy chain (HC)-modified HA in perfused and homogenized lung detected by western blot using IαI antibody (recognizing HC) on lungs before (-) and after (+) hyaluronidase (HAse) liberation of HC linked to HA. Each pair of (-) and (+) lanes represents an individual mouse lung exposed to indicated treatment. Lung HC abundance was determined using densitometry and shown relative to that of total protein. **A**. Lung HC-HA was assessed 12 h after indicated treatments and quantified. **B**. TSG-6's role as the exclusive mediator of HC-HA formation was confirmed using WT and KO lungs. *n* = 4 mice per treatment group. Data analyzed with ANOVA and Tukey's multiple comparisons.

To understand the distinct effects of HC-HA formation during systemic vs. lung-localized LPS exposure, we first investigated the induction of lung parenchymal HC-HA in the two models. Since both IP and IT LPS administration caused lung injury, we hypothesized that lung HC-HA formation would be increased in both models, perhaps even more robustly following IT LPS. We assessed HC-HA formation in whole lung tissue, harvested after circulating cells were removed by perfusion with saline. Lung homogenates were exposed *ex vivo* to hyaluronidase, which cleaves HA into disaccharides [18] and thus releases HA-linked HC detectable by western blot. We noted extensive HC release in lungs at 12 h following IP LPS, compared to IP PBS control (Fig. 2**A,** SFig. 1A), suggesting robust HC-HA formation in lung parenchyma. As controls, we measured HC release in lung tissue from *TSG-6* KO mice that received IP LPS and noted no HC when compared to WT control (Fig. 2**B,** SFig. 1B), consistent with the known role of TSG-6 as exclusive mediator of HC-HA formation. When compared to mice injected with IP LPS, mice receiving LPS directly into lungs (IT) showed a similar amount of HC released by hyaluronidase treatment (Fig. 2**A**). Together with the findings of similar whole lung *TNFα* expression between *TSG-6* KO and control (Fig. 1**B and D**), this suggested that lung HC-HA did not critically drive outcomes following either localized or systemic LPS exposure.

### 3.3 Plasma HC-HA and TSG-6 during severe endotoxemia

**Fig. 3:**
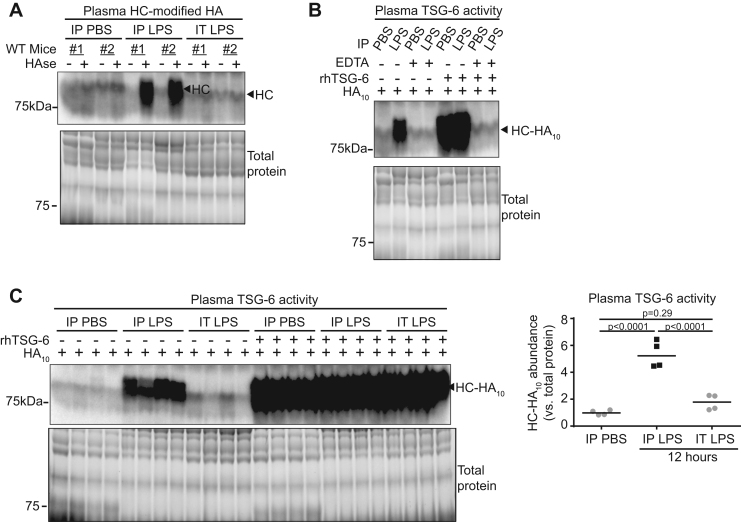
**Plasma HC-HA and TSG-6 induction after IP or IT LPS injury. A**. Abundance of HC-modified HA was detected in plasma collected 12 h after indicated treatments. **B-C**. Endogenous TSG-6 activity in plasma 12 h after indicated treatments was detected by adding 10-oligosacharide HA (HA_10_) and western blot probing with IαI antibody (recognizing HC) for HC linked to HA_10_ (HC-HA_10_). **B**. Recombinant human TSG-6 (rhTSG-6) was used as a positive control to confirm HC-HA_10_ formation and that the sodium citrate anticoagulant used for collecting plasma did not abolish TSG-6 activity. As TSG-6 requires divalent metal ions for activity, excess (0.1 M) EDTA was used as a negative control. **C**. HC-HA_10_ abundance was detected in plasma and quantified using densitometry and expressed relative to total protein. *n* = 4 mice per treatment group. Data analyzed with ANOVA and Tukey's multiple comparisons.

**Fig. 4:**
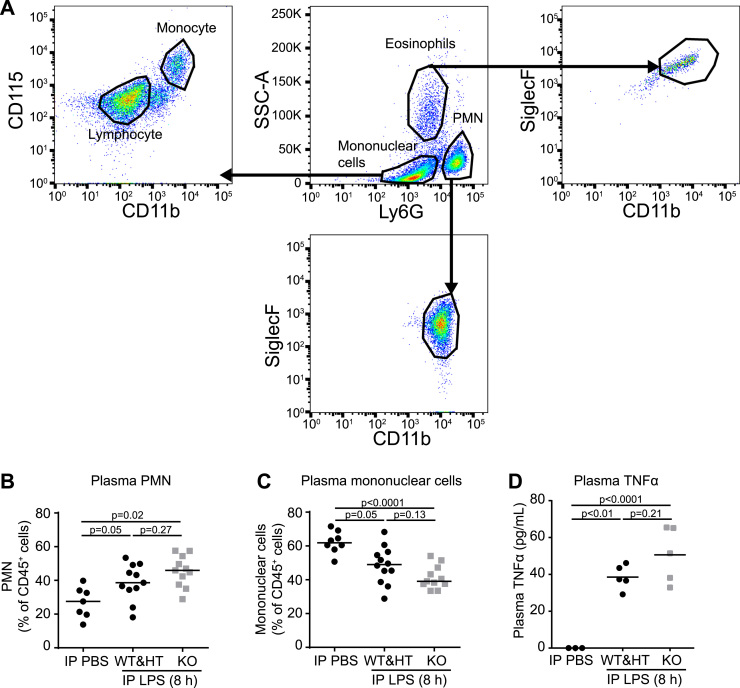
**Effect of TSG-6 on circulating neutrophils and TNFα. A**. Flow cytometry strategy for distinguishing neutrophils (PMN), mononuclear cells, and eosinophils. Hematopoietic cells (depicted in center) were identified using CD45^+^TER-119^-^. PMN were identified as Ly6G^+^SiglecF^-^. Mononuclear cells were identified as SSC^lo^Ly6G^-^SiglecF^-^. Eosinophils were identified as SSC^hi^Ly6G^-^SiglecF^+^. **B-D**. Plasma from *TSG-6* KO and littermate control (WT & HT) mice was collected 8 h after IP LPS or PBS injection. Plasma was analyzed for PMN (**B**) and mononuclear cells (**C**), expressed as percentage of CD45^+^ cells, and for TNFα cytokine levels assessed by ELISA (**D**). *n* = 3–11 mice per treatment group. Data analyzed with ANOVA and Tukey's multiple comparisons.

When compared with controls, the intravascular HC-HA measured in the circulating plasma fraction was induced only after IP LPS, but not after IT LPS administration (Fig. 3**A,** SFig. 2A). Since circulating HA undergoes rapid turnover in the liver sinusoids with a half-life of 2.5–5 min [19], [20], HC-HA would not significantly accumulate unless it was continuously generated. Thus, such marked induction of intravascular circulating plasma HC-HA suggested robust and persistent endogenous TSG-6 secretion in the circulation. To determine if the presence of intravascular HC-HA was associated with increased circulating TSG-6 enzyme activity, plasma was incubated with 10-oligosaccharide HA (HA_10_), and HC-modified HA_10_ was detected by western blot. As positive control, we used excess recombinant TSG-6 (Fig. 3**B,** SFig. 2B). Since TSG-6 mediated HC-modification of HA requires divalent metals Ca^2+^ and Mg^2+^ [13], [14], [15], we used as negative control samples incubated with excess of the irreversible divalent metal chelator EDTA (Fig. 3**B,** SFig. 2B). When compared to IP PBS control, we found significant induction of intravascular TSG-6 activity following IP LPS, but not after IT LPS (Fig. 3**C,** SFig. 2C), suggesting that only systemic LPS exposure causes intravascular HC-HA formation. To substantiate that the model of systemic LPS exposure used caused significant inflammation, we measured circulating inflammatory cells and levels of TSG-6 inducing cytokine TNFα (Fig. 4). When compared to IP PBS control, the percentage of circulating neutrophils (Ly6G^+^ / CD45^+^) of total white blood cells and levels of plasma TNFα were increased following IP LPS administration in control (WT or HT) mice (Fig. 4**B-D**). Although not powered to detect the effect of TSG-6 on sepsis outcomes, we found that these markers of systemic inflammation tended to be higher and percentage of circulating mononuclear cells lower in *TSG-6* KO mice (Fig. 4**B-D**). These results suggested that only systemic, but not lung-localized, LPS exposure induced plasma HC-HA formation and TSG-6 secretion.

### 3.4 Microvascular endothelial TSG-6 expression and secretion

**Fig. 5:**
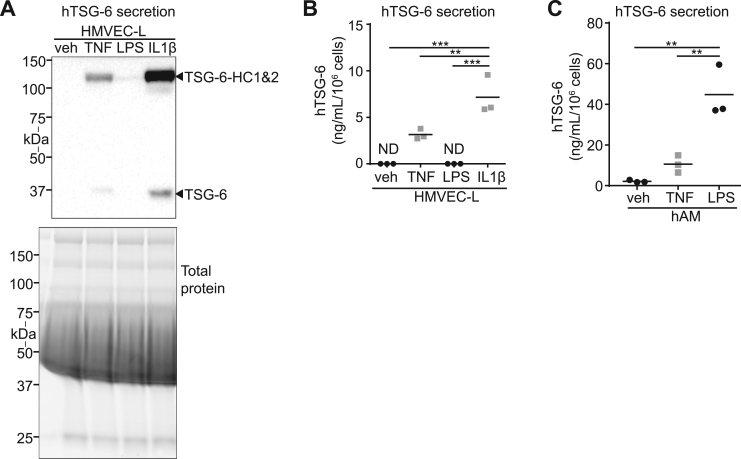
**Inflammatory induction of lung microvascular endothelial TSG-6 secretion. A-B**. Levels of TSG-6 in conditioned media of human lung microvascular endothelial cells (HMVEC-L) after treatment (24 h) with vehicle, TNFα, LPS, or IL1β (20 ng/mL each) were assessed by TSG-6 western blot (**A**) and ELISA (**B**). Levels of TSG-6 secretion (**C**) in primary human alveolar macrophages (hAM) were measured by ELISA. hAM were treated (24 h) with vehicle, LPS (50 ng/mL), or TNFα (20 ng/mL). Not detected (ND). *n* = 3 independent experiments. Data analyzed with ANOVA and Tukey's multiple comparisons. * *P* < 0.05, **P < 0.01, *** *P* < 0.001.

Circulating cells of myeloid origin are likely cellular sources of TSG-6 secretion into circulation during sepsis [10], [21], [22], However, the ability of endothelial cells to secrete TSG-6 in response to inflammatory stimuli is unknown. To address this knowledge gap, primary human lung microvascular endothelial cells (HMVEC-L) were stimulated with LPS or inflammatory cytokines (TNFα or IL1β), which are known to be increased during endotoxic shock [23]. Using western blot (Fig. 5**A**) and ELISA (Fig. 5**B**) of conditioned media, we noted that while LPS did not directly induce TSG-6 secretion from lung endothelial cells, both TNFα and IL1β enhanced TSG-6 secretion. In particular, IL1β potently stimulated the secretion of TSG-6 (35 kDa) and the presence of the covalent intermediate TSG-6-HC (120 kDa, Fig. 5**A**). Since cells are maintained in media containing FBS, a source of bovine IαI used by secreted TSG-6 to remove an HC [10], the presence of the covalent intermediate TSG-6-HC indicates not only secretion of TSG-6, but also functionally active TSG-6 [14]. As expected [10], myeloid cells secreted TSG-6 in response to both TNFα and LPS (Fig. 5**C**). These results implicate endothelial cells as sources of active TSG-6 secretion in the circulation.

## 4 Discussion

Our studies are the first to describe the intravascular induction of TSG-6 enzyme and HC-modified HA during IP LPS-induced endotoxic shock but not during IT LPS-induced ALI. In contrast, both IP and IT LPS-induced injuries induced lung parenchymal HC-HA. These findings suggest that intravascular HC-HA induction may be a key mediator of TSG-6's protective survival effects during endotoxic shock and help explain TSG-6's modest role during localized ALI. In support of this, we found that inflammatory cytokines associated with sepsis stimulate cultured microvascular endothelial cells to secrete TSG-6 that can contribute to intravascular HC-HA formation.

Our finding that TSG-6 promotes survival against IP LPS-induced endotoxic shock is consistent with published studies that *TSG-6* and *IαI* KO mice exhibit greater mortality after IP LPS-induced endotoxic shock [5], [6]. We expanded on previous reports that focused exclusively on lung tissue and differences in lung macrophage polarization [5] to present novel evidence that systemic IP LPS-induced endotoxic shock is associated with intravascular induction of TSG-6 and HC-HA. Intravascular HC-HA is strategically positioned to have protective effects against systemic inflammation due to its role in liver sinusoids, which have the highest intravascular concentration of HA [7], for retaining neutrophils during systemic LPS or gram-negative *E. coli* exposure [8], [9]. While the role of retaining neutrophils in the hepatic sinusoids during bacteremia remains unclear, the sinusoidal vasculature is a key location for neutrophil extracellular trap (NET) release [24], which contributes to systemic bacterial clearance. Additionally, resident sinusoidal macrophages known as Kupffer cells clear damaged and apoptotic neutrophils [25], which may minimize off-target neutrophil-mediated vascular and end-organ damage to protect against endotoxic shock.

What remains unclear is the contribution of TSG-6 and HC-HA in the bone marrow to promoting survival during endotoxic shock. We and others have previously shown that exogenous TSG-6 supplementation can improve hematopoietic progenitor cell function after cigarette smoke-induced myelosupression [16] and alter bone marrow stromal cell differentiation [26], [27], respectively. Additionally, a recent study revealed that CD44-HA binding interactions promote bone marrow progenitor proliferation and increase engraftment following irradiation [28]. Taken together, these findings emphasize the need for future studies to investigate the potential contributions of TSG-6 and HC-HA in the bone marrow during systemic inflammatory conditions that stress and mobilize the bone marrow compartment.

As an inflammation-induced enzyme, TSG-6 is minimally expressed at the transcript level and not detected as a secreted protein during homeostatic conditions [29]. The inflammatory cytokines TNFα and IL1β are potent inducers of TSG-6 secretion in non-hematopoietic cells, whereas both TNFα and LPS induce TSG-6 secretion in myeloid cells [10], [21], [22]. However, while RNA evidence of endothelial TSG-6 has been noted [21], the ability of endothelial cells to secrete TSG-6 had not been reported. Our finding that cultured primary HMVEC-L secrete TSG-6 when stimulated with TNFα or IL1β, but not LPS is consistent with previous reports that showed similar responses in other non-hematopoietic cells [10], [22]. Therefore, during systemic inflammation, microvascular endothelial cells together with circulating myeloid cells may contribute to the intravascular circulating plasma TSG-6 secretion and HC-HA formation. We have not directly measured endothelial glycocalyx-bound HC-HA, an important pool of intravascular HC-HA, which was previously visualized in liver sinusoids using intravital microscopy [8]. Interestingly, since endotoxic shock causes thinning of glycocalyx and releases glycosaminoglycans, including HA, into circulation [30], [31], the circulating HC-HA we detected in plasma may also contain shedded, previously glycocalyx-bound HC-HA.

The TSG-6-mediated HC modification of HA is strategically positioned in the extracellular matrix, from a spatial and temporal standpoint, to signal and direct inflammatory responses [1], [2], [32]. HC-HA has the capacity to bind and to interact with many cellular effectors of inflammation, because the HA receptor CD44 is either constitutively expressed or inducible on most hematopoietic cells [33]. The findings that all HA fragments, from high molecular weight to 8-oligosacharides, can be readily modified with the large HC protein [34] and that in sepsis HA fragments of all sizes are increased in the circulation [35] suggests that the intravascular glycocalyx and circulating HA may undergo dramatic changes during endotoxic shock. While the functional implications of these changes remain to be defined, they may be linked to IαI HC chains’ ability to regulate the complement pathway [36], [37] and neutrophil activation [38]. The covalent modification of high and low molecular weight HA in circulation may serve as a scaffold that attracts CD44 receptor expressing cells to bind HA and undergo HC-mediated effects.

The presence of TSG-6 in serum from patients with bacterial sepsis was noted as unpublished observation [39] and supports the clinical relevance of our novel finding of intravascular TSG-6 and HC-HA induction in an experimental sepsis model. The precise mechanisms by which intravascular modification of HA with HC of the serum protein IαI exerts protective effects in sepsis remain to be elucidated. Such an understanding could lead to the development of therapeutic approaches to ameliorate this prevalent condition that remains a major cause of morbidity and mortality following infection.
