# Supplementary material for: Intravascular heavy chain-modification of hyaluronan during endotoxic shock
Source: Biochem Biophys Rep. 2018 Dec 26;17:114–21. doi: 10.1016/j.bbrep.2018.12.007 (PMC6307094; doi:10.1016/j.bbrep.2018.12.007)
Supplement: Supplementary file 2 — Supplementary material [file mmc2.docx]

**Supplemental Figures**

**Figure S1**. **Entire western blots and total protein controls for Fig. 2.**

**A-B**. Western blots used to generate **Fig. 2A-B**, respectively. Immunoblotting was performed using IαI antibody and total protein loaded in each lane was detected using Stain-Free technology. Besides detecting 75 kDa HC released by hyaluronidase digest of HC-HA in the lung, the IαI antibody detected serum-derived proteins IαI (250 kDa), Pre-α-inhibitor (PαI, consisting of bikunin and HC3, 125 kDa), and possibly TSG-6-HC1&2 (120 kDa), which is difficult to distinguish from PαI.

**Figure S2**. **Entire western blots and total protein controls for Fig. 3.**

**A-C**. Western blots used to generate **Fig. 3A-C**, respectively. Immunoblotting was performed using IαI antibody and total protein loaded in each lane was detected using Stain-Free technology. Note the detection of 75 kDa HC released by hyaluronidase digest of plasma HC-HA (**A**) and HC linked to exogenous HA_10_ for the TSG-6 activity assay (**B-C**). The IαI antibody also detected serum-derived proteins IαI (250 kDa), Pre-α-inhibitor (PαI, consisting of bikunin and HC3, 125 kDa), and possibly TSG-6-HC1&2 (120 kDa), which is difficult to distinguish from PαI.
