# Supplementary figures and images for: Intravascular heavy chain-modification of hyaluronan during endotoxic shock
Source: Biochem Biophys Rep. 2018 Dec 26;17:114–21. doi: 10.1016/j.bbrep.2018.12.007 (PMC6307094; doi:10.1016/j.bbrep.2018.12.007)

# Supplemental Figure 1

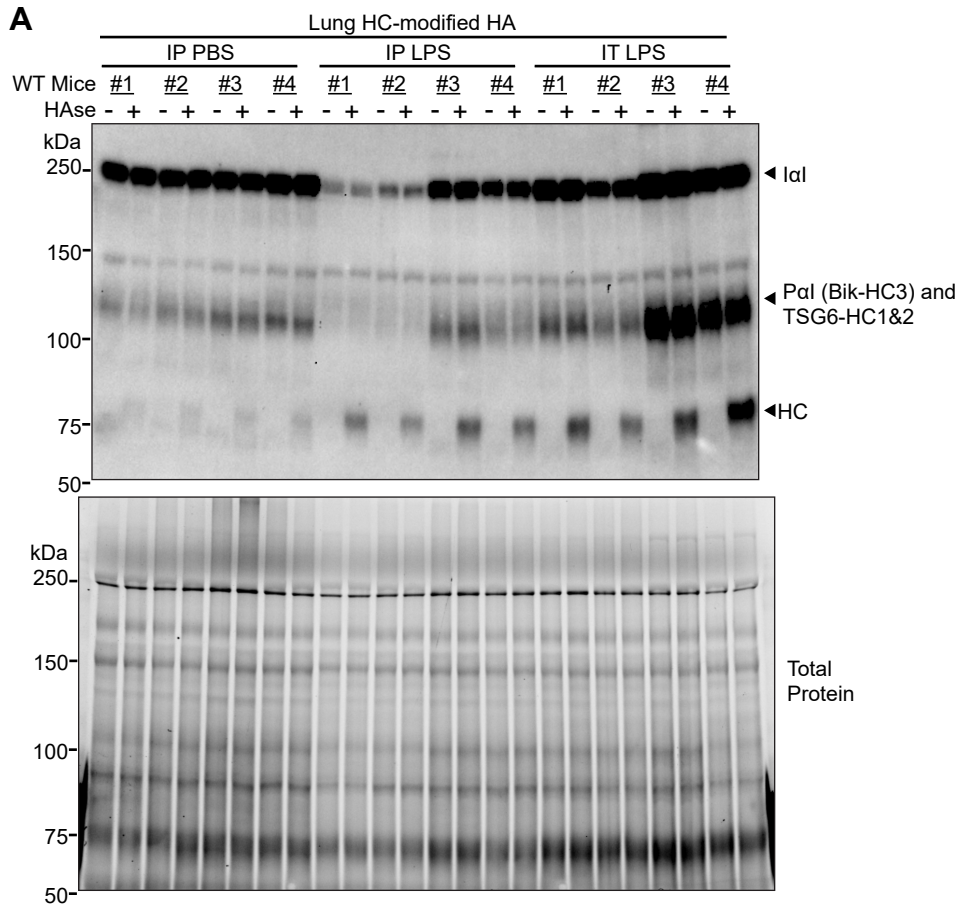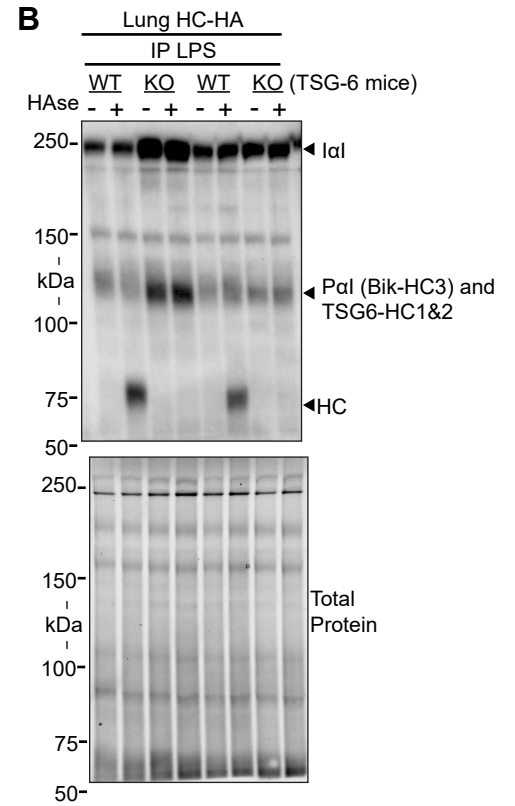

# Supplemental Figure 2

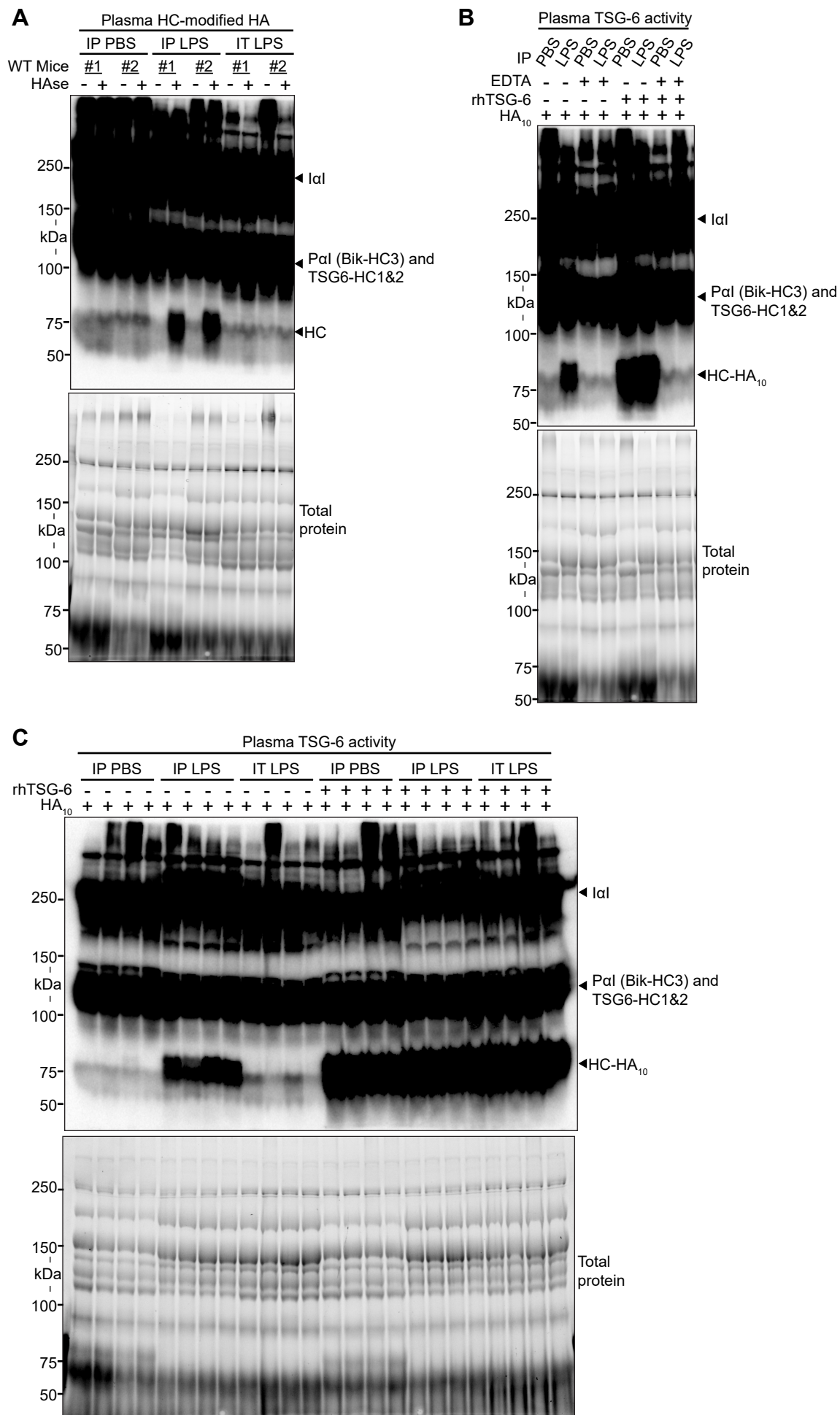

Supplement: Supplementary file 3 — Supplementary material [file mmc3.pdf]
